# The association between breast cancer risk factors and background parenchymal enhancement at dynamic contrast-enhanced breast MRI

**DOI:** 10.1177/0284185120911583

**Published:** 2020-03-26

**Authors:** Roxanna Hellgren, Ariel Saracco, Fredrik Strand, Mikael Eriksson, Ann Sundbom, Per Hall, Paul W Dickman

**Affiliations:** 1 Department of Medical Imaging, Division of Breast Imaging, Södersjukhuset, Stockholm, Sweden; 2 Department of Medical Epidemiology and Biostatistics, Karolinska Institutet, Stockholm, Sweden; 3 Department of Oncology, Södersjukhuset, Stockholm, Sweden; 4 Department of Thoracic Radiology, Karolinska University Hospital, Stockholm, Sweden

**Keywords:** Magnetic resonance imaging, breast, primary neoplasms, epidemiology

## Abstract

**Background:**

Background parenchymal enhancement (BPE) of normal tissue at breast magnetic resonance imaging is suggested to be an independent risk factor for breast cancer. Its association with established risk factors for breast cancer is not fully investigated.

**Purpose:**

To study the association between BPE and risk factors for breast cancer in a healthy, non-high-risk screening population.

**Material and Methods:**

We measured BPE and mammographic density and used data from self-reported questionnaires in 214 healthy women aged 43–74 years. We estimated odds ratios for the univariable association between BPE and risk factors. We then fitted an adjusted model using logistic regression to evaluate associations between BPE (high vs. low) and risk factors, including mammographic breast density.

**Results:**

The majority of women had low BPE (84%). In a multivariable model, we found statistically significant associations between BPE and age (*P* = 0.002) and BMI (*P* = 0.03). We did find a significant association between systemic progesterone medication and BPE, but due to small numbers, the results should be interpreted with caution. The adjusted odds ratio for high BPE was 3.1 among women with density D (compared to B) and 2.1 for density C (compared to B). However, the association between high BPE and density was not statistically significant. We did not find statistically significant associations with any other risk factors.

**Conclusion:**

Our study confirmed the known association of BPE with age and BMI. Although our results show a higher likelihood for high BPE with increasing levels of mammographic density, the association was not statistically significant.

## Introduction

Background parenchymal enhancement (BPE) at dynamic contrast-enhanced breast magnetic resonance imaging (DCE-MRI) is the proportion of enhancing normal fibro-glandular tissue of the breast. The American College of Radiology Breast Imaging-Reporting and Data System (BI-RADS) Atlas 5th Edition classifies BPE into four categories (minimal, mild, moderate, and marked) according to the proportion of fibro-glandular tissue that enhances. BPE usually has a symmetric distribution between the two breasts and has slow early and persistent delayed kinetic features (1). BPE is generally higher in premenopausal women (2). It is hormone sensitive and varies during different phases of the menstrual cycle (3–5), and decreases with tamoxifen treatment (6,7) as well as salpingo-oophorectomy (8). Using a computerized method for measuring absolute BPE in square centimeters, Brown et al. (9) demonstrated that for every 1 cm^2^ reduction in visceral adipose tissue, BPE was reduced by 3.43 cm^2^ (*P* = 0.010), suggesting an association between BPE and body mass index (BMI).

There have been conflicting results regarding whether BPE is an independent risk factor for breast cancer. Several studies of high-risk women, with a cross-sectional study design, have shown increased odds of having cancer with increasing levels of BPE (10–13). A study by Arasu et al. (14), with a median follow-up time of 2.8 years from index MRI to a diagnosis of cancer, showed that compared with minimal BPE, increasing BPE levels were associated with increased cancer risk with hazard ratio of 1.80 for mild, 2.42 for moderate, and 3.41 for marked BPE. A study of high-risk, relatively young women (median age = 44 years) and a median time of three years between baseline MRI and cancer diagnosis found that BPE was not predictive of breast cancer development (15). One study that excluded high-risk women (16) found no association between BPE and breast cancer.

The association between BPE and other risk factors for breast cancer has not been fully investigated. A risk factor of particular relevance is mammographic density, which measures the amount of radio-opaque fibro-glandular tissue on the mammogram. It is well established that women with the highest level of mammographic density have an increased risk of developing breast cancer compared to women with the lowest level of density (17–19). All previous studies of the association between BPE and mammographic density have included women with newly diagnosed breast cancer, and the results cannot automatically be generalized to a healthy population. To our knowledge, only the study by Uematsu et al. (20) has shown a statistically significant positive correlation between background enhancement and mammographic density. All other studies have concluded that there is no significant correlation between BPE and mammographic density (21–24).

The lack of MRI examinations in a healthy cohort with average risk of breast cancer causes difficulties in the interpretation and generalizability of previous results. The unique feature of our study is that the participants were recruited from a cohort study of healthy women, none of whom were known to be high risk. Additionally, we had unique access to information on many risk factors for breast cancer through this prospective cohort study. We performed MRI on a subset of 214 asymptomatic participants. We used these MRI examinations to measure the degree of BPE and studied its association with other established risk factors for breast cancer.

## Material and Methods

### Study population

Between the years 2014 and 2016, 1727 participants aged 40–74 years attending the national, population-based biennial screening program at Södersjukhuset Hospital, Stockholm, Sweden were recruited to participate in a study to test the accuracy of a three-dimensional infrared imaging (3DIRI) prototype (25,26). Women with a positive 3DIRI score and negative screening mammography underwent additional MRI examination within 1–4 weeks from the mammogram with adjustment for the right phase of the menstrual cycle. None of the participants had a known high risk of developing breast cancer, based on family history or genetic predisposition, as high-risk women undergo special surveillance outside the population-based screening program. Women with the following conditions were excluded from the study: the lowest degree of breast density (Volpara percent density <6%) on the previous mammogram; earlier cancer; earlier breast surgery or biopsy <6 weeks before mammography; and ongoing pregnancy. The study was approved by the Regional Ethical Review Board. All participants provided informed consent for the use of the database for future research.

Of 1727 women, 219 (13%) had a negative mammography and a positive 3DIRI result. These 219 women underwent an MRI examination for verification. Five (2%) women had an MRI-detected malignancy. The remaining 214 women, with no detected malignancy, were followed up for one year and none of them had a diagnosis of breast cancer. These women comprise the study population for the present study.

### Mammography

Digital mammography examinations were performed using Philips microdose system (Philips Healthcare, The Netherlands) Amsterdam with breasts examined in the craniocaudal and mediolateral oblique views. All examinations were performed at a single clinic and images were stored in the clinic’s local archive.

### MRI technique

DCE-MRI of the breast was performed according to the guidelines by the European Society of Breast Imaging (27). In premenopausal women, MRI was performed on days 7–14 of the menstrual cycle. Participants underwent MRI in the prone position using a 1.5-T Magnetom Aera (Siemens Medical Solutions, Erlangen, Germany) device with a dedicated 16-channel breast coil and a standard MRI protocol. Intravenous gadolinium contrast media (Dotarem; Guerbet, Villepinte, France) was administered at the dose of 0.2 mL/kg as a bolus injection, with injector, followed by 15 mL of saline solution.

### BPE measurements

**Fig. 1.**
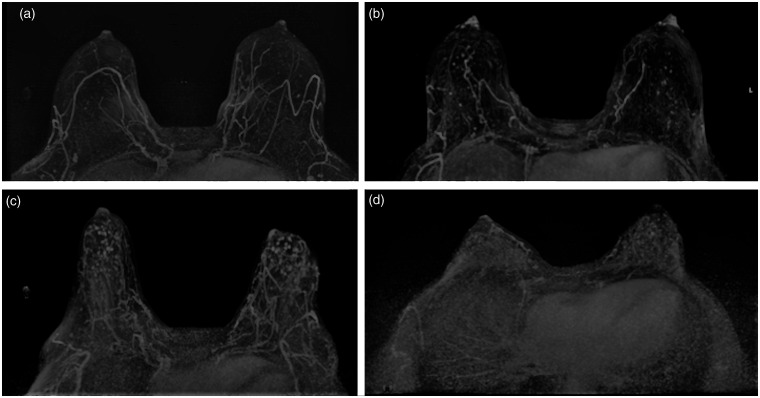
Classification of background parenchymal enhancement according to BI-RADS.

Scores were based on the first post-contrast subtracted fat-suppressed images and the software generated maximum intensity projection (MIP) images. In cases of BPE asymmetry between the left and right breasts, the higher level of BPE was recorded. The degree of BPE was estimated by visual assessment into two categories: low BPE (BI-RADS lexicon 5th edition categories minimal (a) and mild (b)) and high BPE (BI-RADS lexicon 5th edition categories moderate (c) and marked (d)) (Fig. 1).

### Mammographic density measurements

Density was categorized by visual assessment according to the breast density BI-RADS lexicon 5th edition four composition categories: A = the breasts are entirely fatty; B = there are scattered areas of fibro-glandular density; C = the breasts are heterogeneously dense which may obscure small masses; and D = the breasts are extremely dense which lowers the sensitivity of mammography. If there was a difference in the density of the two breasts, the higher level of density was recorded.

Density measurements on the previous mammogram for determination of eligibility for participation in the study was performed using the automated software program Volpara (28).

### Readers

Two radiologists with 8 and 20 years of experience in mammography and 1 year of experience in breast MRI, and one radiology resident with considerable experience in mammography and MRI, performed BPE and density assessments. The final score was decided in consensus.

### Data

Data on age, menopausal status, height, and weight were collected from the case report file, and had been self-reported only a few days before the MRI examinations.

Information on ongoing hormone replacement therapy (local estrogen therapy, systemic estrogen therapy, or systemic combination therapy) or use of systemic contraception containing progesterone was gathered from the case report file data and medical records. In six cases where up-to-date information was not available in our records, the information was collected by contacting the women by telephone.

Data on remaining covariates were retrieved from the cohort study database. The data were collected two years before MRI examinations through a self-reported questionnaire.

Continuous variables such as age, BMI, age at first birth, age at menarche, parity, intake of alcohol, and physical activity were arranged into categories.

### Statistics and data analysis

All explanatory variables were modelled as categorical variables. We first estimated odds ratios (OR) for the univariable associations between BPE and each of the explanatory variables. We then fitted an adjusted logistic regression model to evaluate associations between BPE (classified as high vs. low) and density, age, local estrogen treatment, systemic estrogen treatment (tablets and patches), systemic progestogen treatment (combination hormone replacement therapy as well as contraceptives), BMI, age at menarche, age at first birth, parity, oral contraceptive use, family history of breast cancer, alcohol, smoking, and physical activity. We used likelihood ratio tests to assess the statistical significance of each categorical variable.

Stata version 14.1 (StataCorp, College Station, TX, USA) was used for all statistical analysis.

## Results

A total of 214 women (mean age = 58 years, age range = 43–74 years) were included in the study. The main characteristics of the study participants are listed in Table 1. Of the 214 study participants, 56% had high mammographic density, whereas only 16% had high BPE. In all, 49 women used hormone medication (39 women used only local treatment containing estrogens, five women used systemic estrogen replacement therapy, and five women used preparations containing progesterone). Of the five women using progesterone medication, three were postmenopausal using hormone replacement therapy and two were premenopausal using contraceptives containing progesterone.

**Table 1.** Characteristics of the study participants.

Variables	n (%)
Density (BI-RADS)	
B	94 (44)
C	88 (41)
D	32 (15)
Missing	0
BPE	
Low	180 (84)
High	34 (16)
Missing	0
Age (years)	
40–52	74 (34)
53–62	55 (26)
63–74	85 (40)
Missing	0
Menopausal status	
Pre/perimenopausal	70 (33)
Postmenopausal	144 (67)
Missing	0
Local estrogen	
No	175 (82)
Yes	39 (18)
Missing	0
Systemic estrogen	
No	209 (98)
Yes	5 (2)
Missing	0
Systemic progesterone	
No	209 (98)
Yes	5 (2)
Missing	0
BMI (kg/m^2^)	
<25	155 (72)
25–30	50 (23)
>30	9 (4)
Missing	0
Age at menarche (years)	
<13	79 (37)
13–14	98 (46)
≥15	32 (15)
Missing	5 (2)
Age at first birth (years)	
<26	62 (29)
26–30	37 (17)
>30	73 (34)
Missing	4 (2)
Parity	
Nulliparous	38 (18)
1	37 (17)
≥2	135 (63)
Missing	4 (2)
Oral contraceptive use	
Never	27 (13)
Ever	179 (84)
Missing	8 (4)
Family history of breast cancer	
No	172 (80)
Yes	34 (16)
Missing	8 (4)
Alcohol (g/day)	
<1	49 (23)
1–15	109 (51)
>15	50 (23)
Missing	6 (3)
Smoking	
Never	108 (50)
Former	80 (37)
Current	20 (9)
Missing	6 (3)
Physical activity (43+ MET h/day)	
Low (<43)	113 (53)
High (>43)	93 (43)
Missing	8 (4)
Malignancy	
No	214 (100)
Yes	0
Missing	0

BMI, body mass index; BPE, background parenchymal enhancement.

Table 2 gives the distribution of variables in categories of low versus high BPE along with ORs and 95% confidence intervals (CI) for both the univariable and the multivariable models. We observed a statistically significant inverse association between age and BPE in both the adjusted and unadjusted models. Compared to women aged <52 years, women age 52–62 years had an OR of 0.1 (95% CI = 0.04–0.5) and women aged >62 years had an OR of 0.1 (95% CI = 0.03–0.3) in the unadjusted model and OR of 0.2 (95% CI = 0.04–0.6) and 0.1 (95% CI = 0.02–0.4) in the adjusted model. As expected, we also observed a statistically significant inverse association between BPE and menopausal status with postmenopausal women having an OR of 0.08 (95% CI = 0.03–0.2) of having high degree of BPE compared to premenopausal women. We also found that women using systemic progesterone had an OR of 23.9 (95% CI = 2.6–221) of having high degree of BPE compared to non-users. We also found a statistically significant association between BPE and BMI in the adjusted model. Compared to women with a BMI < 25 kg/m^2^, women with BMI > 30 kg/m^2^ were 4.8 times more likely to have high degree of BPE (95% CI = 1.2–19.4) in the unadjusted model and 12.6 times more likely after adjusting (95% CI = 1.9–82.7). We found a trend for increasing likelihood of having high BPE with increasing levels of density both in the adjusted and the unadjusted models with ORs of 1.7 and 2.1, respectively for BIRADS category C compared to B and 3.3 and 3.1, respectively, for BIRADS category D compared to B. The *P* value, however, was above the 0.05 threshold (0.07 in the unadjusted model and 0.23 in the adjusted model). We did not find any significant associations between high BPE and the remaining risk factors for breast cancer (Table 2).

**Table 2.** Number and percentage of participants with low and high BPE along with ORs of being in the high (vs. low) category from unadjusted and adjusted logistic regression models.

	Low BPE	High BPE	OR unadjusted (95% CI)	OR adjusted (95% CI)
	n (%)	n (%)		
Density (BI-RADS)			*P* = 0.07	*P* = 0.23
B	84 (89)	10 (11)	1.0 (ref)	1.0 (ref)
C	73 (83)	15 (17)	1.7 (0.7–4.1)	2.1 (0.7–6.3)
D	23 (72)	9 (28)	3.3 (1.2–9.0)	3.1 (0.7–12.9)
Age at MRI (years)			*P* < 0.001	*P* = 0.002
0–52	48 (65)	26 (35)	1.0 (ref)	1.0 (ref)
52–62	51 (93)	4 (7)	0.1 (0.05–0.5)	0.2 (0.04–0.6)
>62	81 (95)	4 (5)	0.1 (0.03–0.3)	0.1 (0.02–0.4)
Menopausal status			*P* < 0.001	
Pre/perimenopausal	43 (61)	27 (39)	1.0 (ref)	
Postmenopausal	137 (95)	7 (5)	0.08 (0.03–0.2)	
Local estrogen			*P* = 0.06	*P* = 0.49
No	143 (82)	32 (18)	1.0 (ref)	1.0 (ref)
Yes	37 (95)	2 (5)	0.2 (0.06–1.0)	0.5 (0.09–3.2)
Systemic estrogen			*P* = 0.80	*P* = 0.98
No	176 (84)	33 (16)	1.0 (ref)	1.0 (ref)
Yes	4 (80)	1 (20)	1.3 (0.1–12.3)	1.0 (0.07–15.3)
Systemic progesterone			*P* = 0.01	*P* = 0.03
No	179 (86)	30 (14)	1.0 (ref)	1.0 (ref)
Yes	1 (20)	4 (80)	23.9 (2.6–220.9)	21.1 (1.3–346.0)
BMI (kg/m^2^)			*P* = 0.08	*P* = 0.03
<25	133 (86)	22 (14)	1.0 (ref)	1.0 (ref)
25–29.9	42 (84)	8 (16)	1.2 (0.5–2.8)	1.4 (0.4–4.8)
30+	5 (56)	4 (44)	4.8 (1.2–19.4)	12.6 (1.9–82.7)
Age at menarche (years)			P = 0.18	P = 0.82
<13	62 (78)	17 (22)	1.0 (ref)	1.0 (ref)
13–14	87 (89)	11 (11)	0.5 (0.2–1.1)	0.7 (0.2–2.2)
15+	27 (84)	5 (16)	0.7 (0.2–2.0)	1.0 (0.2–4.6)
Age at first birth (years)			P = 0.09	P = 0.81
Nulliparous	32 (84)	6 (16)	1.0 (ref)	1.0 (ref)
<26	55 (89)	7 (11)	0.7 (0.2–2.2)	1.8 (0.3–10.0)
26–30	35 (95)	2 (5)	0.3 (0.1–1.6)	1.0 (0.1–8.4)
>30	56 (77)	17 (23)	1.6 (0.6–4.5)	1.8 (0.4–7.6)
Parity			*P* = 0.95	
Nulliparous	32 (84)	6 (16)	1.0 (ref)	
1	32 (86)	5 (14)	0.8 (0.2–3.0)	
2+	114 (84)	21 (16)	1.0 (0.4–2.6)	
Oral contraceptive use			*P* = 0.23	*P* = 0.27
Never	25 (93)	2 (7)	1.0 (ref)	1.0 (ref)
Ever	149 (83)	30 (17)	2.5 (0.6–11.2)	3.0 (0.4–20.5)
Family history of breast cancer			*P* = 0.71	*P* = 0.75
No	146 (85)	26 (15)	1.0 (ref)	1.0 (ref)
Yes	28 (82)	6 (18)	1.2 (0.5–3.2)	1.2 (0.4–3.9)
Alcohol (g/day)			P = 0.47	P = 0.70
<1	40 (82)	9 (18)	1.0 (ref)	1.0 (ref)
1–15	91 (83)	18 (17)	0.9 (0.4–2.1)	1.3 (0.4–4.3)
15+	45 (90)	5 (10)	0.5 (0.2–1.6)	0.8 (0.2–3.3)
Smoking			P = 0.90	P = 0.95
Never	90 (83)	18 (17)	1.0 (ref)	1.0 (ref)
Former	69 (86)	11 (14)	0.8 (0.4–1.8)	1.0 (0.4–2.8)
Current	17 (85)	3 (15)	0.9 (0.2–3.3)	0.7 (0.1–4.5)
Physical activity (MET h/day)			*P* = 0.86	*P* = 0.80
Low (<43)	95 (84)	18 (16)	1.0 (ref)	1.0 (ref)
High (>43)	79 (85)	14 (15)	0.9 (0.4–2.0)	0.9 (0.3–2.4)

The adjusted model is adjusted for all variables for which ORs are provided. *P* values are for the likelihood ratio test of general heterogeneity (i.e. comparing models with and without the variable).

BMI, body mass index; BPE, background parenchymal enhancement; CI, confidence interval; MRI, magnetic resonance imaging; OR, odds ratio.

## Discussion

In the present study, we found statistically significant associations between high levels of BPE and age and BMI. Although we did not find a statistically significant association between BPE and mammographic breast density, we did find that the likelihood of having high degree of BPE increased with increasing density levels with a borderline significant *P* value of 0.07 in the unadjusted model. Only 16% of participants had high levels of BPE, while 56% had high mammographic density. Our results suggest that the vascular characteristics of breast tissue, measured by BPE, is to a large extent independent from the amount of fibro-glandular tissue, measured by mammographic density.

Our study supports the conclusion by Hansen et al. (22) that ‘In the majority of women, scores for background enhancement in MRI will be lower than the respective mammographic density scores.’ When performing a histologic examination of the contralateral breast of cancer patients, Sung et al. (29) found that higher levels of BPE were strongly correlated with high levels of microvessel density and glandular concentration in premenopausal women. It is likely that dense breasts of an older population contain low levels of glandular tissue and thus do not show much enhancement. Our study confirms the earlier finding of the positive association between BPE and BMI. Gillman et al. (30) studied 573 women with breast cancer and found that higher BMI was associated with higher BPE. In the WISER Sister study, Brown et al. (31) demonstrated that a reduction of visceral adipose tissue resulted in a significant reduction of BPE. The underlying mechanism for high BPE in obesity is not fully understood. The process could be a result of high levels of endogenous estrogen in obese women. Brooks et al. (32) found that women with high levels of BPE had significantly higher serum levels of estrogen metabolites. In our study, however, we could not find an association between exogenous estrogen prescription and BPE. However, we do not know if women with exogenous estrogen prescription actually took the medication or if taking the medication, they were able to metabolize estrogen to its active metabolites. We speculate that the high level of BPE in obesity could result from an inflammatory milieu in the breasts caused by several inflammatory pathways (33) produced by obese adipocytes (34) even though the fat tissue in itself does not enhance on MRI. Progesterone has a positive association with BPE in our study; however, our result should be regarded with caution, since only five women were using systemic progesterone and we have results with very wide confidence intervals. The mechanism can be progesterone’s effect on vascular permeability (35). Among premenopausal woman, the optimal time for an MRI examination is in the second week of the menstrual cycle before the rise in progesterone levels (27). Several studies have shown that BPE is lowest at this period of the menstrual cycle (3,4,36).

When measuring BPE and density by visual assessment, there is a risk of inter-reader variability and some cases could be misclassified. In order to minimize misclassification, the assessments were performed by three experienced breast radiologists and the final score based on consensus. Our density assessments were similar to the expected proportion for each density category (37). Also, because of the small study sample and lack of power, true but weak associations may not have shown statistical significance. Our study sample consisted mostly of Caucasian, urban women all of whom were attending screening and volunteered to participate in the study. In addition, women with a mammographic density <6% using Volpara had already been excluded from the study. Therefore, our results may not be generalizable to other populations. It could be that our study population differs from a random sample of screened Swedish women because they were selected based on a negative screening mammography and positive 3DIRI result. However, the prevalence of breast cancer among our 219 women was similar to the study by Kuhl et al. (38) where asymptomatic screening women were randomly selected for an MRI examination in addition to mammography. The greatest strength of our study is that, thanks to the cohort study database, we have had access to information on a more complete list of breast cancer risk factors as opposed to many previous studies which have only investigated a limited number of risk factors. Most studies on BPE have been carried out by radiologists with limited information (mainly age, mammographic density, and BMI) and on women clearly not representative of a healthy, average-risk population.

Although DCE-MRI remains the standard for breast cancer diagnosis, there is a move towards MRI sequences that do not use contrast due to potential side effects of gadolinium. The most clinically used sequences are diffusion-weighted images (DWI) and measurement of apparent diffusion coefficient (ADC) values. A recently published meta-analysis shows that ADC measurements have a pooled sensitivity of 89% and specificity of 82% for breast cancer (39). Kawamura et al. (20) have shown that ADC values of normal breast tissue have a significant inverse correlation with BPE. In the current study, however, we have not measured ADC values as it is not yet an established risk factor for breast cancer.

In conclusion, the likelihood of having high levels of BPE is increased with young age, high BMI, and progesterone medication. We did not find a statistically significant association between BPE and mammographic density although we found a trend of increasing likelihood for having high level of BPE with increasing levels of density categories.
